# Genome wide single cell analysis of chemotherapy resistant metastatic cells in a case of gastroesophageal adenocarcinoma

**DOI:** 10.1186/1471-2407-11-455

**Published:** 2011-10-20

**Authors:** Geir Olav Hjortland, Leonardo A Meza-Zepeda, Klaus Beiske, Anne H Ree, Siri Tveito, Hanne Hoifodt, Per J Bohler, Knut H Hole, Ola Myklebost, Oystein Fodstad, Sigbjorn Smeland, Eivind Hovig

**Affiliations:** 1 Oslo University Hospital, Division for Cancer and Surgery, Department of Oncology, The Norwegian Radium Hospital, P.O. Box 4950, Nydalen, N-0424 Oslo, Norway; 2 Oslo University Hospital, Institute for Cancer Research, Department of Tumor Biology, The Norwegian Radium Hospital, P.O. Box 4950, Nydalen, N-0424 Oslo, Norway; 3 Oslo University Hospital, Department of Pathology, Rikshospitalet, P.O. Box 4950, Nydalen, N-0424 Oslo, Norway; 4 Oslo University Hospital, Department of Pathology, The Norwegian Radium Hospital, P.O. Box 4950, Nydalen, N-0424 Oslo, Norway; 5 Oslo University Hospital, Department of Medical Imaging and Intervention, The Norwegian Radium Hospital, P.O. Box 4950, Nydalen, N-0424 Oslo, Norway; 6 Oslo University Hospital, Section for Medical Informatics, The Norwegian Radium Hospital, P.O. Box 4950, Nydalen, N-0424 Oslo, Norway; 7 Institute for informatics, University of Oslo, Oslo, Norway

## Abstract

**Background:**

Metastatic progression due to development or enrichment of therapy-resistant tumor cells is eventually lethal. Molecular characterization of such chemotherapy resistant tumor cell clones may identify markers responsible for malignant progression and potential targets for new treatment. Here, in a case of stage IV adenocarcinoma of the gastroesophageal junction, we report the successful genome wide analysis using array comparative genomic hybridization (CGH) of DNA from only fourteen tumor cells using a bead-based single cell selection method from a bone metastasis progressing during chemotherapy.

**Case presentation:**

In a case of metastatic adenocarcinoma of the gastroesophageal junction, the progression of bone metastasis was observed during a chemotherapy regimen of epirubicin, oxaliplatin and capecitabine, whereas lung-, liver and lymph node metastases as well as the primary tumor were regressing. A bone marrow aspirate sampled at the site of progressing metastasis in the right iliac bone was performed, and single cell molecular analysis using array-CGH of Epithelial Specific Antigen (ESA)-positive metastatic cells, and revealed two distinct regions of amplification, 12p12.1 and 17q12-q21.2 amplicons, containing the KRAS (12p) and ERBB2 (HER2/NEU) (17q) oncogenes. Further intrapatient tumor heterogeneity of these highlighted gene copy number changes was analyzed by fluorescence in situ hybridization (FISH) in all available primary and metastatic tumor biopsies, and ErbB2 protein expression was investigated by immunohistochemistry.

ERBB2 was heterogeneously amplified by FISH analysis in the primary tumor, as well as liver and bone metastasis, but homogenously amplified in biopsy specimens from a progressing bone metastasis after three initial cycles of chemotherapy, indicating a possible enrichment of erbB2 positive tumor cells in the progressing bone marrow metastasis during chemotherapy. A similar amplification profile was detected for wild-type KRAS, although more heterogeneously expressed in the bone metastasis progressing on chemotherapy. Correspondingly, the erbB2 protein was found heterogeneously expressed by immunohistochemical staining of the primary tumor of the gastroesophageal junction, while negative in liver and bone metastases, but after three initial cycles of palliative chemotherapy with epirubicin, oxaliplatin and capecetabine, the representative bone metastasis stained strongly positive for erbB2.

**Conclusion:**

Global analysis of genetic aberrations, as illustrated by performing array-CGH analysis on genomic DNA from only a few selected tumor cells of interest sampled from a progressing bone metastasis, can identify relevant therapeutic targets and genetic aberrations involved in malignant progression, thus emphasizing the importance and feasibility of this powerful tool on the road to more personalized cancer therapies in the future.

## Background

The incidence of adenocarcinomas of the gastric cardia, the gastroesophageal junction (GEJ) and esophagus has had a sharp rise in the past few decades, in particular among white males in the United States and western Europe, whereas the incidence of gastric cancer of the corpus or pyloric region has declined in many western countries [1-3]. In contrast to squamous cell carcinomas of the esophagus, which are strongly associated to alcohol and tobacco consumption, adenocarcinomas of the lower esophagus, GEJ and proximal stomach are typically associated with factors like Barrett's esophagus, gastroesophageal reflux, asthma medications, cholecystectomy, and obesity [4-9]. The dismal outcome of these patients, often due to the advanced disease stage at diagnosis, underlines the need for new treatment options, as well as better understanding of the biology of the disease. Improving therapy of metastatic gastroesophageal adenocarcinoma is warranted. A landmark study in this regard was the recently published randomized phase III ToGA study [10], establishing c-erbB2 (HER-2/neu) as a target for therapy in advanced gastric and gastroesophageal junction cancer, with clinically significant improved overall survival for patients treated with trastuzumab in a first line regimen of 5-fluorouracil/capecitabine and cisplatin. As gastric cancer reveals more heterogeneity in the expression of erbB2 than breast cancer, a modified scoring system for gastric cancer was agreed upon at an international consensus meeting [11], and used by the panel of pathologists in the ToGA study. Patients having tumors with high expression of erbB2 (IHC3+) and ERBB2 FISH positive revealed a particular benefit of the trastuzumab treatment, whereas the benefit was less for the patients with lower expression levels of erbB2 or with negative FISH. However, in light of these exciting data, the clinical significance of intrapatient erbB2 heterogeneity in metastatic gastroesophageal cancer at diagnosis and during ongoing chemotherapy is less well known. Different metastatic tumor cell clones may show different sensitivity to chemotherapy, or change during disease progression, due to metastatic evolution [12]. In this case of adenocarcinoma of the gastroesophageal junction, we examined the erbB2 expression in multiple metastatic lesions as well as the interlesional heterogeneity in response to chemotherapy. The chemotherapy resistant metastatic cells in a progressing metastatic bone lesion after the initial cycles of chemotherapy were analyzed genome-wide by screening of DNA copy number changes to identify oncogenic aberrations and potential targets of therapy.

## Case presentation

**Figure 1:**
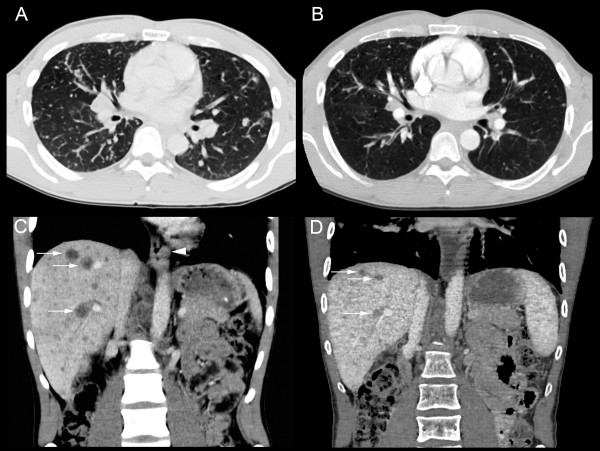
**CT scans before and after the first three cycles of chemotherapy**. (A) Chest CT scan at the time of diagnosis revealing multiple metastases in both lungs, and (B) the corresponding image after chemotherapy. (C) Coronal section from an abdominal CT scan at the time of diagnosis showing multiple liver metastases (three are marked with arrows) and also the tumor of the left wall of the gastroesophageal junction (arrowhead). (D) Corresponding section as C in an abdominal CT scan after chemotherapy showing decreased size of liver metastasis.

A 35-year old white male, non-smoker, was admitted to hospital as he had experienced increasing pain in the upper part of the abdomen, and his general practitioner had discovered elevated levels of serum transaminases. He had no previous medical record, except appendectomy. During the previous 2-3 months, he had lost 3-4 kgs of weight and had experienced episodes of night sweat, but no fever, and a varying degree of edema in both legs. On physical examination, he appeared slim, ECOG (Eastern Cooperative Oncology Group) performance status 0. The abdomen was normally configured, but the liver was palpable 3 cm below the costal ridge. He had a slight edema of both legs and ultrasonic examination showed thrombosis of deep veins distally of the popliteal fossae in both lower extremities. Colonoscopy was normal, but gastroscopy revealed a non-stenotic small polypous lesion in the gastroesophageal junction, 34 cm from the dental lining and 3 cm above the *linea dentata*. CT scans showed multiple metastases (Figure 1) in both lungs and several pathologically enlarged lymph nodes up to 2 cm in diameter in the mediastinum, both lung hili, the liver hilus and in the retroperitoneal compartment. There were multiple metastases in all liver segments, and the left side of the distal esophageal wall had a thickened appearance. MRI of the head, spine and pelvis revealed bone metastases scattered in several locations of the spine and pelvis. Laboratory investigations showed blood values within reference limits of differentiated blood cell counts, electrolytes, creatinine, urea, glucose, bilirubin and albumin. There were elevated serum levels of lactate dehydrogenase (568 U/L; normal range < 205 U/L), alkaline phosphatase (650 U/L; normal range < 105 U/L), alanine aminotransferase (174 U/L; normal range 10-70 U/L), carcinoembryonic antigen (CEA;1833 μg/L), CA-125 (2747 kU/L) and MUC-1 (115 kU/L).

Biopsies from the gastroesophageal tumor and metastases in the liver and bone marrow were sampled. The histopathology revealed a moderately differentiated adenocarcinoma, with glandular structures formed by atypical columnar epithelium, with unevenly and aberrantly distributed chromatin structures within the cell nuclei, and tumor infiltration of normal squamous epithelium. Immunohistochemical staining was positive for cytokeratin (CK) 7, caudal related homeobox-2 (CDX-2), CEA, and negative for CK20, prostate specific antigen, synaptophysin, chromogranin A, and thyroid transcription factor TTF-1, thus supporting the diagnosis of a neoplasm of the gastroesophageal junction as the primary tumor.

**Figure 2:**
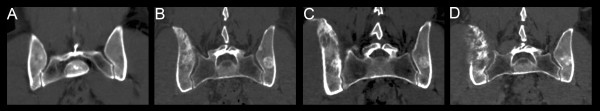
**CT scans, coronal sections, showing growth of bone metastasis at different time points during the progression of the disease**. (A) CT scan at the time of diagnosis, (B) after 9 weeks (C) after 16 weeks, and (D) after 24 weeks.

**Figure 3:**
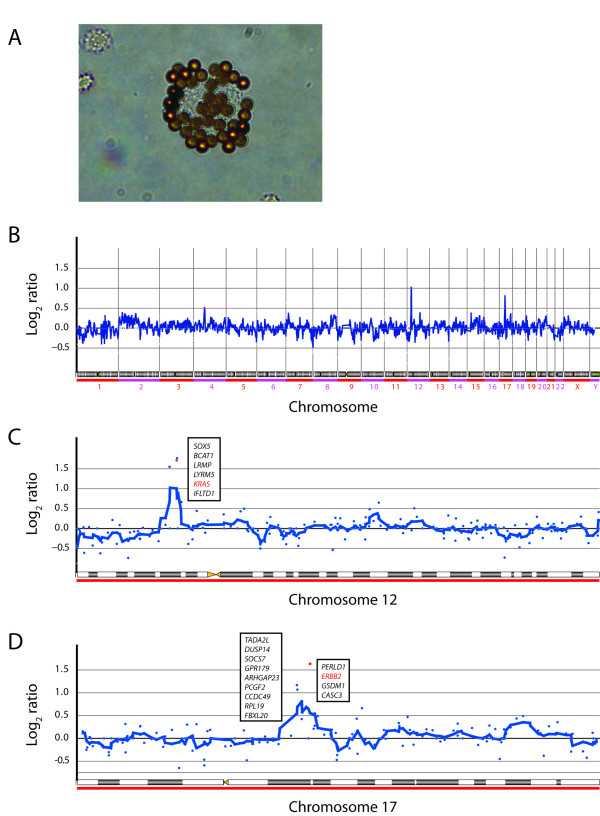
**Tumor cells from bone marrow aspirate and the results from array CGH analysis performed on such tumor cells**. (A) An ESA positive tumor cell rosetted with magnetic beads conjugated with an anti-ESA antibody. (B) Array CGH data of 14 ESA-positive tumor cells displaying low resolution of amplification levels of all 23 chromosomes extended. (C) A close-up of the 12p amplicon, and (D) close-up of the 17q amplified locus.

**Figure 4:**
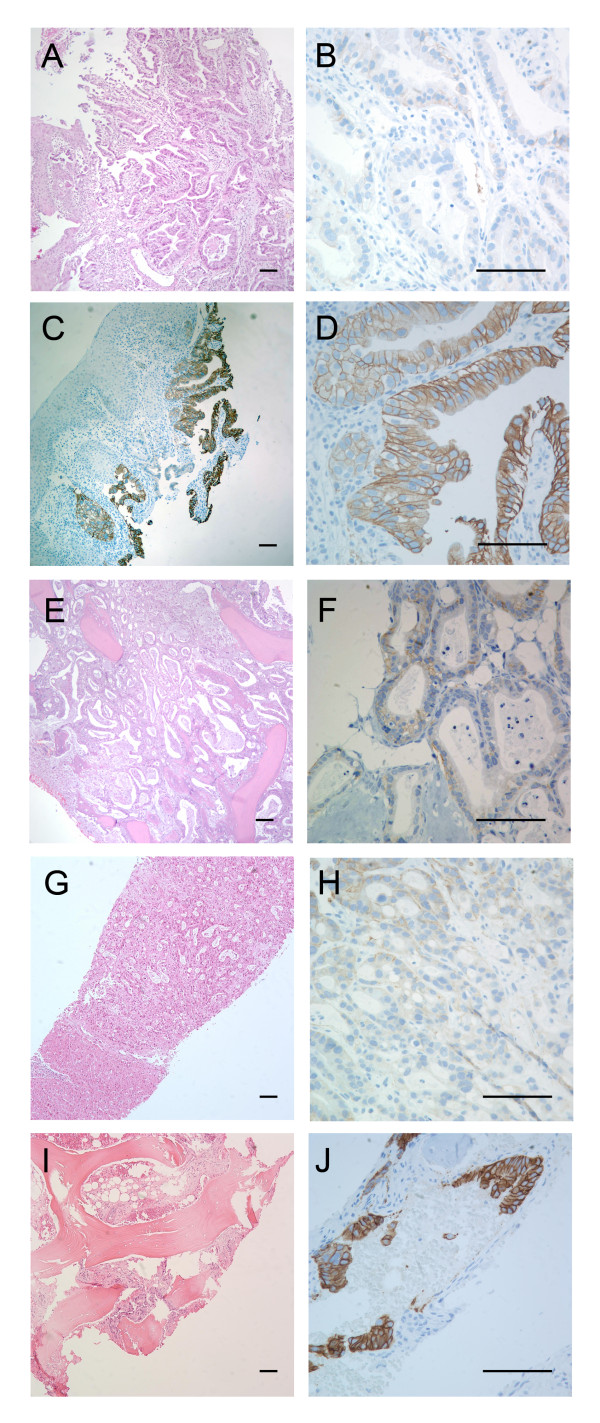
**Biopsies obtained from the primary oesophageal tumor, liver metastasis and right iliac bone metastasis of the 35-year-old male at the time of diagnosis, and from a corresponding right iliac bone metastasis after three cycles of chemotherapy**. (A) Hematoxylin and eosin (HE) staining of the primary oesophageal tumor and corresponding immunohistochemical c-erbB2 staining (brown) of the same biopsy (B-D), where both positive and negative tumor cells are seen (C). (B) A close-up of C showing erbB2 negative tumor cells. (D) A close-up of C showing erbB2 positive tumor cells. (E) HE staining of a bone biopsy sampled at the time of diagnosis. (F) erbB2 staining of the same bone biopsy as E, showing only weakly positive staining (1+) and termed clinically negative. (G) HE staining of a liver metastasis biopsy. (H) erbB2 staining of the same liver metastasis biopsy, showing only weakly positive staining (1+) and termed clinically negative. (I) HE staining of a biopsy from bone metastasis of the right iliac bone sampled after three cycles of chemotherapy. (J) erbB2 immunohistochemical staining of the same biopsy as I, showing bone marrow infiltrated with tumor cells strongly positive (3+) for erbB2.

**Figure 5:**
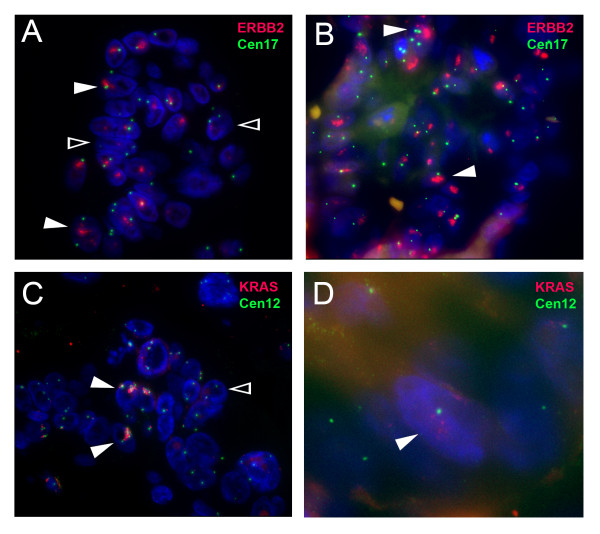
**ERBB2 and KRAS gene amplification**. Open arrows indicate examples of non-amplified cells, whereas closed arrows point to cells with gene amplification. Images were taken with a Zeiss Axioplan 2 fluorescence microscope objective ×60. (A) Fluorescence in situ hybridization of the ERBB2 gene (red) in the primary gastroesophageal tumor shows amplified signals as compared to the reference centromere 17 (green). (B) FISH analysis of ERBB2 (red) and centromere 17 (green) in an iliac bone metastasis progressing during chemotherapy. (C) FISH analysis of the KRAS gene (red) and centromere 12 (green) in the primary gastroesophageal tumor. (D) FISH analysis of KRAS (red) and centromere 12 (green) in an iliac bone metastasis progressing during chemotherapy. One positive cell is displayed at ×2,5 higher magnification than cells in A-C. Blue color indicates DAPI nuclear counterstaining.

After the first three cycles of palliative chemotherapy with epirubicin 50 mg/m^2 ^and oxaliplatin 130 mg/m^2 ^intravenously every third week and continuous oral administration of capecitabine (Xeloda^®^) 625 mg/m^2 ^twice daily (EOX), thoracic and abdominal CT scans showed reduction of lung, liver and lymph node metastases (Figure 1). Serum CEA was reduced from 2685 μg/L at onset of chemotherapy to 76 μg/L. In contrast, metastases in the pelvic skeleton and spine had progressed (Figure 2), A bone marrow aspirate and a new biopsy was sampled at this point from the right iliac bone for molecular analysis of the tumor cells and a second histological analysis. Fourteen cells rosetted with anti-ESA coated magnetic beads (Dynal^® ^Magnetic Beads, Invitrogen) were picked using a motorized microscope device fitted with a glass capillary (CellPick)[13]. Genomic DNA from these cells was amplified using the Genomplex Single cell WGA kit (Invitrogen) (Figure 3). To identify genomic aberrations, array comparative genomic hybridization (CGH) was performed using a 1 Mb resolution genomic microarray essentially as described previously [14]. Genome-wide analysis of copy number changes identified two distinct regions of amplification, 12p12.1 and 17q12-q21.2, both approximately 3.3 Mb in size. A number of genes are known to be located within the amplified regions, among them KRAS (12p) and ERBB2 (HER2/NEU) (17q). Amplified DNA from the same cells was also subjected to KRAS exon-1 mutation detection [15], and no mutation was identified. As also seen in many gastric adenocarcinomas, immunohistochemical analysis of erbB2 protein expression in the biopsies from the primary gastroesophageal tumor showed heterogeneity, with both negative (1+) cells next to nests of positive cells (3+) (Figure 4). The erbB2 immunohistochemical scoring was performed as described previously [11]. In biopsies from pelvic bone and liver metastases, immunohistochemical staining showed tumor cells negative (1+) for the erbB2 protein, but after the first three cycles of EOX, mainly positive erbB2 (3+) expressing tumor cells were seen in the bone marrow. Amplification of the ERBB2 and KRAS genes were analyzed by FISH, applying commercial probes for ERBB2, centromere 12 and 17 (Abbott Molecular Inc., Des Plaines, IL, USA) and two BAC-clones spanning the chromosome 12p12.1 region (RP11-583L24 and RP11-707G18) for KRAS on the biopsies mentioned above. In the primary tumor, we found that both ERBB2 and KRAS were heterogeneously amplified (Figure 5). For ERBB2 analysis, 270 tumor cell nuclei were evaluated and 66 (24.4%) showed amplification with an average ERBB2/centromere 17 ratio of 6,1. FISH analysis of KRAS and centromere 12 revealed 124 amplified among 450 investigated cells (27.6%) with an average KRAS/centromere 12 ratio of 4,8. Both ERBB2- and KRAS-amplified cells were heterogeneously distributed between non-amplified cells, either as small clusters or as single cells. In the liver metastasis, both KRAS and ERBB2 were heterogeneously amplified, but in the bone metastasis we found no ERBB2 or KRAS amplification. However, in the bone metastasis biopsy sampled after three courses of EOX, ERBB2 was homogenously amplified. Here, 92 of 120 (76.7%) evaluated tumor cell nuclei scored positive with an average ERBB2/Centromere 17 ratio of 7,6. Due to the limited amount of metastatic tumor cells in deeper sections, only 22 nuclei were available for the FISH analysis of KRAS and 8 of these displayed amplification (Figure 5).

Chemotherapy was continued, and after two more cycles of EOX, CT scan evaluation showed a further reduction of the lung, liver and lymph nodes metastases, serum CEA was further reduced to 39 μg/L. CT evaluation also showed bone metastasis progressing steadily and unaffected of the palliative chemotherapy given. Due to increased pain in his lower back and the pelvic area, the patient received palliative radiotherapy to 30 Gy in two weeks to the affected area. The EOX chemotherapy was continued and he received zoledronic acid due to lytic bone lesions. Two additional cycles of EOX was given, but due to general disease progression the treatment was discontinued. He experienced increased pain, had decreased appetite and had lost an additional 5 kgs of weight. Shortly after, he was admitted to hospital because of acute neurological symptoms with pareses and paralysis of left cranial nerves (i.e. the trigeminal (V) and abducens (VI) nerves). An MRI scan revealed multiple meningeal metastatic deposits and disseminated pathologic lesions in the bone marrow of the scull, facial bones and spine. High-dose glucocorticosteroid treatment relieved the symptoms, and he was given palliative radiotherapy (to 30 Gy in two weeks) towards the whole brain, including the meninges and base of the skull. His health condition did not allow further treatment with any second-line chemotherapy regimen, but a regimen of trastuzumab monotherapy (4 mg/kg i.v. followed by 2 mg/kg week i.v.) was initiated. Six weeks thereafter he was admitted to the local hospital because of acute respiratory distress and pneumonia. One week after the hospital admission, the patient deteriorated and died.

## Discussion

A main challenge in metastatic cancer treatment is the development or enrichment of therapy-resistant tumor cells. In this young patient with stage IV gastroesophageal adenocarcinoma, we performed global gene analysis of DNA copy number changes in selected tumor cells sampled from a chemotherapy-resistant bone marrow metastasis. The gene analysis showed amplification of KRAS and ERBB2 oncogenes as possible molecular targets of therapy in tumor cells. However, after the discontinuation of EOX therapy, erbB2 targeted therapy with trastuzumab monotherapy was unable to provide substantial disease control, since his general health condition deteriorated rapidly. Other factors in addition to erbB2 receptor expression and signaling could also be important to sustain disease progression. In the case of cetuximab treatment of colorectal cancer, which targets another receptor of the ERBB-family (the epidermal growth factor receptor) the expression of receptor ligands (i.e. amphiregulin and epiregulin), as well KRAS mutation, predict treatment response [16]. For our patient, we did not detect any mutation in the KRAS gene, but amplification of the gene was detected. Potentially, high expression of the GTPase Kras oncoprotein, due to gene amplification, could sustain oncogenic signaling despite erbB2 inhibition by trastuzumab treatment. Other mechanisms of trastuzumab resistance could also be involved [17]. Immunohistochemical analysis showed heterogeneous expression of erbB2, with distinct erbB2-positive tumor cell populations before chemotherapy in the primary tumor, but negative cells in the liver and bone marrow, with cells staining strongly positive for erbB2 in bone marrow after the three initial cycles of chemotherapy. These results were furthermore supported by the finding of a similar pattern for ERBB2 gene amplification, as shown by the FISH results. Heterogeneous gene amplification was found both for the ERBB2 and the KRAS genes, but particularly enrichment of ERRB2 amplified cells was found in the biopsy from the bone metastasis after the initial three courses of chemotherapy. The clinically validated c-erbB2 immunohistochemistry analyses and ERBB2 FISH analyses supported and served as controls for the array CGH results of ERBB2 gene amplification found in the tumor cells harvested from the separate bone marrow aspirate, indicating an enrichment of such cells in the bone marrow metastasis which clearly progressed during the initial three courses of chemotherapy as shown on the computer tomography radiographs. A decreased sensitivity to the EOX treatment in the ERBB2 amplified cells could be one explanation for the enrichment of such cells during chemotherapy.

The patient died 9.5 months after onset of first line treatment with EOX chemotherapy. Median overall survival for patients with advanced gastroesophageal cancer receiving EOX chemotherapy in the REAL2 study was reported to be 11.2 months [18]. As in breast carcinomas, amplification of ERBB2 is found in 20-30% of adenocarcinomas of the esophagus, gastroesophageal junction and stomach, and is associated with poorer survival [19,20]. Interventional protocols targeting erbB2 in gastroesophageal adenocarcinoma have been conducted [21]. Data from the large phase III ToGA trial was recently published [22], where stage IV c-erbB2 positive gastric or esophageal adenocarcinomas were randomized to receive cisplatin and 5-FU or capecitabine ± trastuzumab, showing a significantly prolonged survival for the trastuzumab arm. These results have introduced c-erbB2 targeted therapy in the first line treatment for selected patients with metastatic gastroesophageal adenocarcinomas. Among several genetic abnormalities, both KRAS and ERBB2 amplification have been indicated as early events in adenocarcinoma development of Barrett's Esophagus [23]. Amplification of wild-type KRAS has been implicated in therapy resistance *in vitro*, but the clinical importance is yet poorly described. Analysis of aberrations at the molecular level found in each patient's tumor cells can identify new therapeutic options. Such molecular characterization of tumors to identify potential therapeutic targets has been performed in pilot studies [24].

## Conclusion

Global analysis of genetic aberrations, as illustrated in this case by performing array-CGH analysis on genomic DNA from only a few selected tumor cells of interest, can by way of identifying resistance markers obtain information on potential therapeutic targets responsible for malignant progression, as well as potential predictors of treatment response, thus emphasizing the importance of this powerful tool on the road to more personalized cancer therapies in the future.

## Consent

Written informed consent was obtained for publication of this case report and any accompanying images. A copy of the written consent is available for review by the Editor-in-Chief of this journal.

## Abbreviations

CGH: comparative genomic hybridization; CA-125: cancer antigen 125; CEA: carcinoembryonic antigen; CT: x-ray computed tomography; ERBB2: v-erb-b2 erythroblastic leukemia viral oncogene homolog 2 (gene) alias HER2/NEU; ESA: epithelial specific antigen i.e. EpCAM: epithelial cell adhesion molecule; FISH: fluorescence in situ hybridization; KRAS: v-Ki-ras2 Kirsten rat sarcoma viral oncogene homolog (gene); MUC1: mucin 1 cell surface associated; MRI: magnetic resonance imaging.

## Competing interests

The authors declare that they have no competing interests.

## Authors' contributions

EH had the initial design of the study, and GOH, AHR, SS were responsible for the diagnosis and treatment of the patient, the writing of the manuscript and data analysis and interpretation of results. LAMZ performed the array CGH analysis, HH and ST isolated and selected tumor cells from bone marrow, KHH was responsible for radiologic images and interpretation, PB was responsible for immunohistochemical analysis of tumor samples and KB performed the FISH analyses. ØF, OM and EH was providing the necessary laboratory facilities, financial support as well as manuscript writing. All authors read and approved the final manuscript.

## Pre-publication history

The pre-publication history for this paper can be accessed here:

http://www.biomedcentral.com/1471-2407/11/455/prepub
